# Voxel-Mirrored Homotopic Connectivity of Resting-State Functional Magnetic Resonance Imaging in Blepharospasm

**DOI:** 10.3389/fpsyg.2018.01620

**Published:** 2018-09-11

**Authors:** Jing Wei, Shubao Wei, Rongxing Yang, Lu Yang, Qiong Yin, Huihui Li, Yuhong Qin, Yiwu Lei, Chao Qin, Jingqun Tang, Shuguang Luo, Wenbin Guo

**Affiliations:** ^1^Department of Neurology, The First Affiliated Hospital of Guangxi Medical University, Nanning, China; ^2^Department of Radiology, The First Affiliated Hospital of Guangxi Medical University, Nanning, China; ^3^Department of Psychiatry, The Second Xiangya Hospital of Central South University, Changsha, China

**Keywords:** blepharospasm, resting state, functional magnetic resonance imaging, voxel-mirrored homotopic connectivity, support vector machine

## Abstract

**Objective:** Several networks in human brain are involved in the development of blepharospasm. However, the underlying mechanisms for this disease are poorly understood. A voxel-mirrored homotopic connectivity (VMHC) method was used to quantify the changes in functional connectivity between two hemispheres of the brain in patients with blepharospasm.

**Methods:** Twenty-four patients with blepharospasm and 24 healthy controls matched by age, sex, and education were recruited. The VMHC method was employed to analyze the fMRI data. The support vector machine (SVM) method was utilized to examine whether these abnormalities could be applied to distinguish the patients from the controls.

**Results:** Compared with healthy controls, patients with blepharospasm showed significantly high VMHC in the inferior temporal gyrus, interior frontal gyrus, posterior cingulate cortex, and postcentral gyrus. No significant correlation was found between abnormal VMHC values and clinical variables. SVM analysis showed a combination of increased VMHC values in two brain areas with high sensitivities and specificities (83.33 and 91.67% in the combined inferior frontal gyrus and posterior cingulate cortex; and 83.33 and 87.50% in the combined inferior temporal gyrus and postcentral gyrus).

**Conclusion:** Enhanced homotopic coordination in the brain regions associated with sensory integration networks and default-mode network may be underlying the pathophysiology of blepharospasm. This phenomenon may serve as potential image markers to distinguish patients with blepharospasm from healthy controls.

## Introduction

Blepharospasm (BSP) is a common clinical type of focal dystonia characterized by involuntary blinking and eyelid spasms (Hallett et al., 2008). BSP is a chronic disease with an incidence rate of 4.2/10 million in general population (Steeves et al., 2012). This disease occurs mostly in adults and manifested with an increased frequency of blinking in the early stage and consistent closed-eyes or even functional blindness in the late stages. Furthermore, BSP will lead to poor quality of life, decline in employment, anxiety, and depression (Muller et al., 2002; Biuk et al., 2013; Valls-Sole and Defazio, 2016; Bedarf et al., 2017). However, the pathophysiology of BSP remains unclear.

Recent techniques, such as neuroimaging, facilitate the exploration of structural and functional abnormalities in patients with BSP. For example, Baker et al. reported regional abnormalities in the cortical and subcortical brain areas in BSP (Baker et al., 2003; Yang et al., 2013). A voxel-based morphometry study demonstrated increased gray matter in the putamen (Etgen et al., 2006) and primary sensorimotor cortex (Suzuki et al., 2011) in patients with BSP. Zhou et al. (Zhou et al., 2013) used the amplitude of a low-frequency fluctuation method and found abnormalities in the bilateral somatosensory regions in patients with BSP.

The above mentioned studies indicate that multiple abnormal nodes are involved in the pathophysiology of BSP. However, the reported findings are inconsistent in regard to specific networks. For example, a report with independent component analysis exhibited decreased connectivity within the sensorimotor network and the right fronto-parietal network but increased connectivity in the salience network in patients with BSP (Huang et al., 2017). By contrast, a functional connectivity study reported that patients with BSP showed altered functional connectivity at rest in widespread brain regions including basal ganglia, cerebellar, primary/secondary sensorimotor, and visual areas (Jochim et al., 2018). Therefore, BSP may be related to network abnormality, and dysfunction of these nodes is likely to be involved in the pathogenesis of BSP. However, whether or not these abnormalities result in simultaneous bilateral eyelid spasms in BSP remain ambiguous. Moreover, complex functional connectivities exist between two cerebral hemispheres, and their effects are complementary and coordinated. Both eyelids simultaneously experience spasms in BSP, and this event is related to abnormalities of functional interaction between two cerebral hemispheres. Thus, BSP might have also resulted from brain connection dysfunction. However, whether or not an abnormal functional interaction exists between two cerebral hemispheres in BSP should be confirmed.

Resting-state fMRI (rs-fMRI) has attracted increasing attention (Biswal et al., 1995), because it can detect patterns of coherent intrinsic activities of the brain and interactions between two hemispheres. Voxel-mirrored homotopic connectivity (VMHC) (Zuo et al., 2010) is a method of rs-fMRI used to analyze functional homotopy between two hemispheres. The synchrony in patterns of spontaneous activity between homotopic regions in each hemisphere is an important feature of the functional structure of the brain (Stark et al., 2008). The VMHC is designed to directly compare the interhemispheric resting-state functional connectivity. This process can also measure the correlations between blood oxygen level-dependent time series and reflect the communication pattern of information between two cerebral hemispheres. Thus, VMHC is extremely important for information integration of the brain. Recent studies used VMHC to show abnormal homotopic connection in patients with schizophrenia (Hoptman et al., 2012; Guo et al., 2014b, 2017a,b) and their unaffected siblings (Guo et al., 2014a), depression (Guo et al., 2013a,b), somatization disorder (Su et al., 2016), and Parkinson’s disease (Hu et al., 2015). Moreover, Anderson et al. reported that homotopic resting-state functional connectivity was disrupted in individuals with autism (Anderson et al., 2011), and this finding indicated that homotopic function is an important part of the brain function. In addition, excision of corpus callosum could damage the sensory, motor, and cognitive functions of human brain. This phenomenon implies that the coordination between two hemispheres plays an important role for human behavior (Luo et al., 2015). The abovementioned studies suggest that abnormal interhemispheric connectivity may be an important factor in the occurrence of BSP. Based on the onset pattern of simultaneous effect on the bilateral eyelids of patients with BSP and the presence of different networks and loop injuries, we hypothesized that hemisphere connectivity might play a key role in the pathogenesis of BSP. Using VMHC to compare the fMRI data of patients with BSP with those of healthy controls, we evaluated spatial heterogeneity of interhemispheric functional connectivity and clarified the pathogenesis of BSP. To date, findings in brain networks of patients with BSP are limited. To our knowledge, the current study is the first work to employ VMHC to investigate the resting-state functional connectivity of the two hemispheres in patients with BSP.

Patients with BSP usually demonstrate psychiatric symptoms, such as anxiety and depression (Muller et al., 2002; Yang et al., 2017). Self-rating Anxiety Scale (SAS) and Self-rating Depression Scale (SDS) were used to evaluate the symptoms of anxiety and depression, respectively, to control the confounding impact from these conditions. Prior to the rating, all patients were screened to determine whether they were suffering from anxiety or depression. Moreover, Jankovic Rating Scale (JRS-S) was used to assess the severity of eyelid spasm. Then, The VMHC values of different brain areas were correlated with the degree of JRS-S, the course of disease, and the SAS and SDS scores to examine the relationship between VMHC values and clinical symptoms. Finally, SVM was employed to examine whether abnormal VMHC values could discriminate patients from controls with high accuracy and specificity.

## Materials and Methods

### Subjects

We recruited 26 outpatients with BSP who visited the Department of Neurology in the First Affiliated Hospital of Guangxi Medical University between November 2012 and June 2014. The inclusion criteria for the BSP group were as follows: (1) met the criteria of BSP diagnosis according to the Clinical Guidelines of BSP (Defazio et al., 2013); (2) absence of structural changes with conventional MRI examination; (3) had not used botulinum toxin within 3 months prior to the study; (4) had not used medication for dystonia within 1 month prior to the study; and (5) right-handedness. Patients with the following conditions were excluded: (1) secondary BSP from other diseases, such as hepatolenticular degeneration and dry eye; and (2) history of neurological and psychiatric disorders.

The following information was collected from patients with BSP: sex, age, education level, course of disease, degree of illness severity, scores of SAS, and scores of SDS. JRS-S was used to assess the severity of BSP. SAS and SDS were used to examine the severity of anxiety and depression.

Twenty-four healthy volunteers from the community were recruited as age- sex-, and education level-matched controls. All individual controls were right-handed and had no history of severe neuropsychiatric diseases, medical illness, or family history of neurological or psychiatric disorders from the first-degree relatives.

All participants read and signed a consent form before the examination. The Ethics Committee of the First Affiliated Hospital, Guangxi Medical University approved this study.

### Image Acquisition

Data were acquired using a German Siemens Trio Tim 3.0T scanner (Erlangen, Germany). Head fixers and earplugs were used for all subjects to reduce head movement and machine noise, respectively. The subjects were required to remain motionless, awake, and eye-closed during image acquisition. All patients underwent routine examination (T1W1 and T2W1) to exclude intracranial lesions. The following parameters were used for functional imaging: repetition time/echo time (TR/TE) = 2000/30 ms, 30 slices, 64 × 64 matrix, 90° flip angle, 24 cm FOV, 4 mm section thickness, 0.4 mm gap, and 250 volume (500 s).

### Data Preprocessing

Images were preprocessed using the data assistant software (DPABI) (Yan et al., 2016). The first 10 time points were removed. All participants had less than 2 mm maximum displacement in the *x*, *y*, or *z-*axis and 2° of angular motion during data acquisition. The images were then normalized to the standard SPM8^1^ echo planar imaging template with resampling to 3 × 3 × 3 mm^3^ voxels. The processed images were smoothed with an isotropic Gaussian kernel (full-width at half-maximum = 8 mm). Finally, the acquired data were subjected to temporal bandpass filtering (0.01–0.08 Hz) and linear detrending to reduce the effect of low-frequency drifts and physiological high-frequency noise. Spurious covariates and their temporal derivatives, including Friston-24 head motion parameters, white matter signals, and cerebrospinal fluid signals, were removed from the data using linear regression.

### Interhemispheric Correlation and Statistical Analysis

The software REST^2^ was used to analyze VMHC, and the details of VMHC computation has been expounded in a previous study (Zuo et al., 2010). Individual VMHC maps were generated for each participant by computing Pearson correlation (Fisher *z*-transformed) between a given voxel and a corresponding voxel in the contralateral hemisphere. Correlation values were then Fisher *z*-transformed to improve the normality. The resultant values were applied for group comparisons and generation of the VMHC maps.

Individual-level VMHC maps were analyzed using group-level voxel wise *t*-test to determine regional group differences in VMHC. The significance level was set to *p* < 0.05 for multiple comparisons corrected by the Gaussian Random Field theory (voxel significance: *p* < 0.001, cluster significance: *p* < 0.05). Given that resting-state functional connectivity could be influenced by micro motions (Power et al., 2012), the frame wise displacement values were computed as a covariate for each subject in the group comparisons. Pearson correlation was used to evaluate the relationships between VMHC values with significant group differences and degree of JRS-S, SAS scores, SDS scores, and the course of disease after the normality of these variables being assessed.

The demographic and clinical information was compared with the two-sample *t*-test and *χ*^2^-test. The significance level was set to *P* < 0.05.

### Classification Analysis Using SVM

SVM was conducted to evaluate the possibility of abnormal VMHC in these clusters to discriminate patients from healthy controls using the LIBSVM software package^3^ in MATLAB (Chang and Lin, 2011). The LIBSVM classifier was trained by providing examples of the form <x, c>, where x represents the VMHC values of these abnormal clusters, and c is the class label (*c* = +1 for patients with BSP and *c* = −1 for healthy controls). A sample set was divided into a training set and a test set for SVM to evaluate the classification performance on the unobserved data. The leave-one-pair-out method was applied for the LIBSVM classifier algorithm. We constructed a random SVM cluster based on the brain fMRI data of the subjects for classification and feature selection. To acquire the optimal sensitivity and specificity, default functional kernels of Gaussian radial basis and the grid search method were applied to optimize the parameters with the “leave-one-subject-out” method.

## Results

### Demographics and Clinical Characteristics of the Participants

A total of 24 patients were included in the further analysis (two patients were excluded due to excessive head movement). About 20.83 and 29.17% of patients with BSP had anxiety and depressive symptoms, respectively. Additionally, 3 (12.5%) patients with BSP had comorbidity of depression. Moreover, 19 patients with BSP (79.16%) had sensory tricks. These patients might temporarily improve eyelid spasms by wearing glasses, having material in the mouth, or touching the cheeks, forehead, or jaw. Half of the 24 patients (50.00%) experienced worsened eyelid spasms when talking. The patient and control groups did not differ significantly in sex, age, and education levels (**Table 1**).

**Table 1:** Clinical profile of the participants.

	BSP patients (*n* = 24)	Healthy controls (*n* = 24)	*p*-value
Sex (male/female)	8/16	6/18	0.53^a^
Age (years)	49.58 ± 8.58	50.88 ± 8.13	0.59^b^
Years of education (years)	10.38 ± 2.34	10.63 ± 2.16	0.70^b^
Degree of JRS-S (1–4 degree)	2.63 ± 0.82		
SAS score	43.79 ± 8.11		
SDS score	47.95 ± 8.58		
Illness duration (months)	11.00 ± 3.82		

*BSP, blepharospasm; SAS, Self-Rating Anxiety Scale; SDS, Self-Rating Depression Scale; JRS-S, Jankovic Rating Scale. ^a^p-value for gender distribution was obtained by Chi-square test. ^b^p-values were obtained by two-sample t-test.*

### VMHC: Group Differences

**FIGURE 1:**
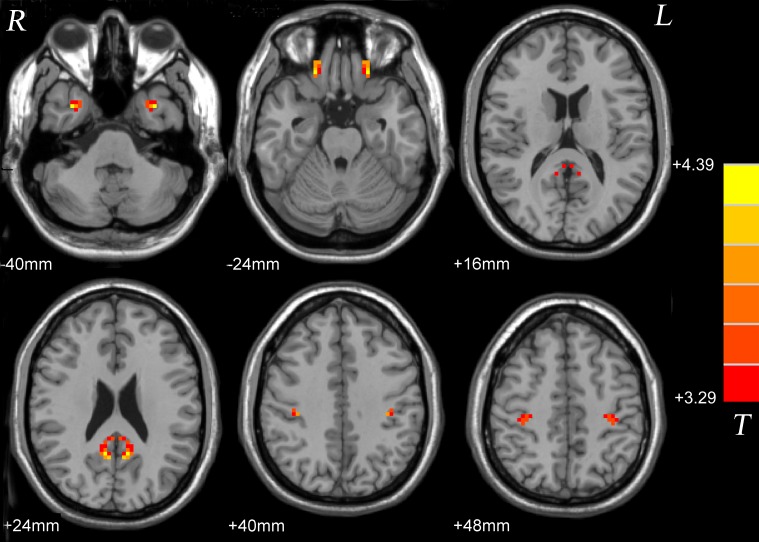
Statistical maps showing VMHC differences between patients and controls. Red denotes increased VMHC, and color bars indicate *T*-values from *t*-tests between groups. VMHC, voxel-mirrored homotopic connectivity.

Compared with the healthy control group, significantly high VMHC was found in the inferior temporal gyrus, interior frontal gyrus, posterior cingulate cortex and postcentral gyrus in the patient group (**Figure 1** and **Table 2**).

**Table 2:** Significant group differences in VMHC.

Cluster location	Peak (MNI)			Cluster size	*T*-value
	*x*	*y*	*z*		
Patients > controls					
Inferior temporal gyrus	±33	6	−39	24	4.2707
Inferior frontal gyrus	±21	33	−24	44	4.2388
Posterior cingulate cortex	±9	−54	24	78	4.3927
Postcentral gyrus	±36	−27	42	76	4.1915

*VMHC, voxel-mirrored homotopic connectivity; MNI, Montreal Neurological Institute.*

### Correlation Analysis

No significant correlation was found between abnormal VMHC values and the severity of symptom, clinical course, and scores of SAS and SDS in the patients (*P* > 0.05).

### SVM for Classification Analysis

**FIGURE 2:**
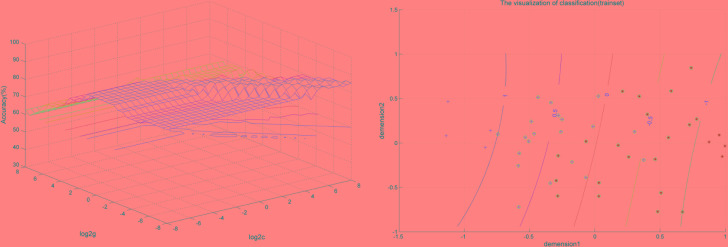
Visualization of classification by support vector machine (SVM) using the combination of the VMHC values in the Inferior Frontal Gyrus and Posterior Cingulate Cortex. Left: SVM parameters selection result (3D visualization) [Grid Search Method]: Best *c* = 0.70711; Best *g* = 0.088388; Right: the visualization of classification with a combination of the VMHC values in the Inferior Frontal Gyrus and Posterior Cingulate Cortex.

**FIGURE 3:**
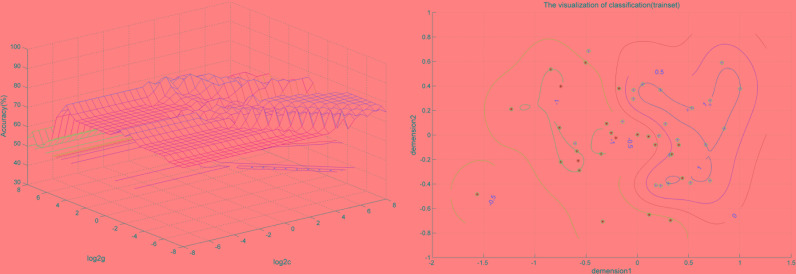
Visualization of classification by support vector machine (SVM) using the combination of the VMHC values in the abnormal brain regions. Left: SVM parameters selection result (3D visualization) [Grid Search Method]: Best *c* = 0.70711; Best *g* = 11.3137; Right: the visualization of classification with a combination of the VMHC values in the Inferior Temporal Gyrus and Postcentral Gyrus.

SVM analysis was performed to determine whether or not abnormal VMHC values could satisfactorily discriminate patients with BSP from healthy controls. The results showed that the VMHC values in a single brain region could not discriminate patients with BSP from healthy controls with optimal sensitivity, specificity, and accuracy (**Table 3**), but the patients may be distinguished with high sensitivity, specificity, and accuracy (more than 80%) using a combination of two brain regions. Among the brain regions, the ability of discriminating patients with BSP from the healthy controls by the combination of the VMHC values in the inferior frontal gyrus and posterior cingulate cortex was optimal with an accuracy of 87.5% (42 of 48 in the 2 groups), a sensitivity of 83.33% (20 of 24 in the BSP group), and a specificity of 91.67% (22 of 24 in the control group). The combination of the VMHC values in the inferior temporal gyrus and postcentral gyrus showed an accuracy of 85.4% (41 of 48 in the 2 groups), a sensitivity of 83.33% (20 of 24 in the BSP group), and a specificity of 87.5% (21 of 24 in the control group; **Figures 2**, **3**). The other combinations such as inferior frontal gyrus/inferior temporal gyrus or posterior cingulate cortex/postcentral gyrus had unsatisfactory sensitivity and specificity.

**Table 3:** Discriminate the patients from the controls by using the VMHC values of a single region with the SVM method.

Brain regions	Sensitivity	Specificity	Accuracy
Inferior frontal gyrus	79.17% (19/24)	75.00% (18/24)	77.08% (37/48)
Inferior temporal gyrus	62.50% (15/24)	87.50% (21/24)	75.00% (36/48)
Postcentral gyrus	58.33% (14/24)	91.67% (22/24)	75.00% (36/48)
Posterior cingulate cortex	70.83% (17/24)	83.33% (20/24)	77.08% (37/48)

*VMHC, voxel-mirrored homotopic connectivity; SVM, support vector machines.*

## Discussion

In this study, higher VMHC values were found in some brain regions, including the inferior temporal gyrus, interior frontal gyrus, posterior cingulate cortex, and postcentral gyrus in patients with BSP than those in healthy controls (*P* < 0.05). Moreover, SVM analysis revealed that the VMHC values in a single brain region could not discriminate patients with BSP from healthy controls, contrary to the combination of two brain regions with high sensitivity, specificity, and accuracy. Moreover, no significant correlation was found between abnormal VMHC values and the severity of symptoms, clinical course, and scores of SAS and SDS in the patient group (*P* > 0.05).

Previous studies indicated that BSP was related to abnormal function of the basal ganglia (Perlmutter et al., 1997; Federico et al., 1998; Esmaeli-Gutstein et al., 1999). Meanwhile, other studies (Martino et al., 2011; Zhou et al., 2013) have reported the abnormalities in the central posterior and posterior cingulate gyrus in patients with BSP. Our report is consistent with those from previous studies (Martino et al., 2011; Zhou et al., 2013). Patients with BSP demonstrated various patterns of sensory tricks, such as motor, imaginary, forcible, and reverse sensory tricks (Hallett, 2002; Ramos et al., 2014), and more likely to suffer from significant prepulse inhibition hand stimulation when presented sensory tricks (Gomez-Wong et al., 1998). One report indicated increased sensory afferents in the eyelid activity control in patients with BSP (Hallett, 2002). Another report implied that the changes in sensory input played an important role in sensory integration abnormalities which might result from increased sensory afferents (Patel et al., 2014). Thus, balancing the movement output and sensory afferents might be a valuable strategy to improve symptoms of eyelid spasm. Furthermore, this report also implied that abnormalities in the sensory center and sensory integration were involved in the pathogenesis of dystonia. Wong et al. reported that 8 of 17 patients with BSP (47.06%) showed sensory tricks (Gomez-Wong et al., 1998). In our patient group, 19 of the 24 BSP patients (79.16%) presented sensory tricks. The eyelid spasms could be alleviated when the patient wears glasses and touches his cheek, forehead, jaw, or other parts of his body. The VMHC of the postcentral gyrus was significantly increased. This phenomenon suggested that patients with BSP had to process more complex information and programs to control eyelid movement. Thus, the integration of sensory-motor information might play an important role in the pathogenesis of BSP. Furthermore, 12 of the 24 BSP patients (50.00%) demonstrated reverse sensory tricks. These patients experienced worse symptoms when they talked (Greene and Bressman, 1998). Interestingly, a stimulus or action can alleviate the symptoms in some patients but not effective in other patients with BSP or even worsening their symptoms (Wider et al., 2004; Martino et al., 2010; Ramos et al., 2014). Thus, a sensory trick has a very unique individual nature. However, the complex input mechanism of sensory integration is unclear, and more studies are needed to explore the related neurophysiological factors in patients with BSP.

The patients in our study presented increased connection from bilateral inferior frontal gyrus, where Brodmann areas 44 and 45 of the inferior frontal gyrus are the main parts of Broca area. Previous study suggested that the frontal lobe plays an integrated role in sensory information processing, and this concept supports the hypothesis that the Broca area is part of the sensory network (Okada et al., 2016). Recent studies showed that the human hyperdirect low-frequency interactions between the prefrontal cortex and subthalamic nucleus (STN) support the regulation of several related brain functions (Kelley et al., 2018), and the STN is the key point of the motor network (Alexander and Crutcher, 1990). In Parkinson’s disease, high frequencies of typical STN deep brain stimulation protocols are used to treat motor symptoms. Therefore, we can speculate that the STN can also become a therapeutic target in the future therapy of BSP.

In 2001, Raichle et al. (Raichle et al., 2001) first proposed default mode network (DMN). This network exhibits high levels of activity at rest. However, DMN becomes deactivated when specific goal-directed behavior is required. DMN consists of the posterior cingulate cortex, precuneus, medial prefrontal cortex, ventral anterior cingulate cortex, inferior parietal lobule, and several temporal lobes (Fransson, 2006). Zhou et al. found abnormalities in the medial prefrontal cortex and posterior cingulate cortex, and these abnormalities suggested significant differences in the DMN in patients with BSP (Zhou et al., 2013). The posterior cingulate cortex, which involves monitoring sensation, stereotactic positioning, and other functions, is the only region in the cingulate cortex that accepts thalamic occipital medial projection (Baleydier and Mauguiere, 1980). A bidirectional connection exists between the pulvinar thalamus and the secondary sensory cortex area (Brodmann areas 37, 39, and 40). The joint fibers connect to the ipsilateral occipital lobe, temporal lobe, frontal lobe, and contralateral brain region. An abnormal homozygous connection was found in the posterior cingulate cortex. This result suggested an abnormal loop between the posterior cingulate cortex and the thalamus and secondary sensory cortex. The posterior cingulate cortex is the intermediate connector between the two hemispheres. Moreover, the bilateral cerebral hemispheres are asymmetric. These findings suggest that the abovementioned changes may be compensatory reactions to the cortical center. Furthermore, the posterior cingulate cortex is an important node as a static state of DMN and might affect the functions and related connections of DMN. Damage in the bilateral temporal lobe of the monkey brain resulted in the degeneration of the fiber that connects the posterior cingulate cortex and the temporal lobe (Papez, 1995). In addition, the temporal gyrus is relevant to the information integration of vision. A study that experimented on color discrimination and visual contrast perception has demonstrated that the visual impairment of patients with BSP was not dependent on the severity of the disease or the course of the disease (Buttner et al., 1999). In the present study, increased VMHC values were found in the posterior cingulate cortex and inferior temporal gyrus, and these values suggested that patients with BSP might have abnormal visual-spatial sensory integration. However, whether or not the posterior cingulate cortex and inferior temporal gyrus are the exact key points of the visual spatial sensory integration remains unclear. The question needs to be confirmed by animal experiments. Our results provide evidence for the establishment of animal models in the future.

No correlation was found between SAS or SDS scores and increased VMHC values in the patients. Three of the 24 patients with BSP were diagnosed with depression in this study. This finding indicated the higher morbidity of depression in patients with BSP than in the normal population (The morbidity of depression in the normal population was approximately 2–6%) (Blazer et al., 1994; Dunlop et al., 2003; Ohayon and Schatzberg, 2003; Gu et al., 2013). Moreover, several scholars inferred that patients with focal dystonia easily suffer from anxiety and depressive symptoms (Voon et al., 2010). SAS and SDS are self-rating scales which might be affected by some confounders, such as educational levels, intelligence, illness duration, and social environment. No correlation was found between abnormal VMHC and anxiety and depression of BSP, which is consistent with a previous study (Stamelou et al., 2012). Furthermore, no correlation was found between abnormal VMHC and the course of disease and severity degrees of BSP symptoms. In previous studies, no difference was found in the age and severity of dystonia between patients with BSP with or without mood disorders (Fabbrini et al., 2010). This phenomenon suggested that mood disorders were not a direct response to focal dystonia (Lencer et al., 2009; Horovitz et al., 2012). Thus, patients with BSP may suffer from anxiety and depression, but these conditions do not interfere with the evolution of the disease. Previous studies reported that changes in the gray matter volume in patients with BSP were not related to illness duration (Martino et al., 2011; Horovitz et al., 2012) and severity degree (Martino et al., 2011) of BSP symptoms. However, another study has shown that abnormal gray matter density in patients with BSP was related to the course of disease (Suzuki et al., 2011). The conflicting results may be due to abnormal sensory motor plasticity (Abbruzzese and Berardelli, 2011) and neuronal remodeling uncertainty (Opavsky et al., 2006).

SVM analyses showed that the VMHC values in one single brain region could not discriminate patients with BSP from healthy controls with optimal sensitivity, specificity, and accuracy, contrary to a combination of two brain regions with high sensitivity, specificity, and accuracy. Meanwhile, the specificity was particularly remarkable, because every healthy control was correctly classified. Therefore, the combination of high VMHC values in the inferior frontal gyrus and posterior cingulate cortex, as well as in the inferior temporal gyrus and postcentral gyrus, may serve as a potential image marker to distinguish the patients with BSP from healthy controls. Our report is consistent with those of fMRI studies in patients with schizophrenia (Li et al., 2018; Wang et al., 2018). Therefore, early detection and improved accuracy of the diagnosis in patients with BSP may be achieved based on the fMRI imaging using the combination of inferior frontal gyrus and posterior cingulate cortex or the inferior temporal gyrus and postcentral gyrus.

This study has several limitations. Patients with BSP demonstrate a wide spectrum of symptoms with different severity degrees and illness durations. However, stratified analysis was not performed because of the small sample size. More patients should be recruited to confirm the present report. We will use stratified analysis to study the relationship between abnormal VMHC values and the symptoms or course of disease. Then, whether or not connection abnormality is a fundamental change or compensatory performance of BSP will be confirmed. The VMHC method reflects the synchrony in patterns of spontaneous activity between homotopic regions in each hemisphere and does not fully reflect the functional state of the whole brain. Therefore, we can simultaneously employ the regional homogeneity method to analyze local consistency changes in the whole brain regions.

## Conclusion

Abnormalities of BSP were found in the inferior temporal gyrus, interior frontal gyrus, posterior cingulate cortex, and post-central gyrus. The results suggested that those related brain areas may be good candidate regions to explore the nature of BSP and highlight the significance of sensory integration and DMN in the pathophysiology of the disorder. Moreover, a combination of high VMHC values in two brain areas can serve as a potential image marker to distinguish patients with BSP from healthy controls.

## Ethics Statement

This study was carried out in accordance with the recommendations of the First Affiliated Hospital of Guangxi Medical University. The protocol was approved by the the Ethics Committee of the First Affiliated Hospital of Guangxi Medical University. All subjects gave written informed consent in accordance with the Declaration of Helsinki.

## Author Contributions

SL and WG designed the study. JW, RY, SW, LY, HL, QY, YL, YQ, JT, and CQ collected the original imaging data. WG, JW, and SW managed and analyzed the imaging data. JW and SW wrote the first draft of the manuscript.

## Conflict of Interest Statement

The authors declare that the research was conducted in the absence of any commercial or financial relationships that could be construed as a potential conflict of interest.

## Footnotes

1 http://www.fil.ion.ucl.ac.uk/spm

2 http://resting-fmri.sourceforge.net

3 http://www.csie.ntu.edu.tw/cjlin/libsvm
